# Full Genome Sequence and sfRNA Interferon Antagonist Activity of Zika Virus from Recife, Brazil

**DOI:** 10.1371/journal.pntd.0005048

**Published:** 2016-10-05

**Authors:** Claire L. Donald, Benjamin Brennan, Stephanie L. Cumberworth, Veronica V. Rezelj, Jordan J. Clark, Marli T. Cordeiro, Rafael Freitas de Oliveira França, Lindomar J. Pena, Gavin S. Wilkie, Ana Da Silva Filipe, Christopher Davis, Joseph Hughes, Margus Varjak, Martin Selinger, Luíza Zuvanov, Ania M. Owsianka, Arvind H. Patel, John McLauchlan, Brett D. Lindenbach, Gamou Fall, Amadou A. Sall, Roman Biek, Jan Rehwinkel, Esther Schnettler, Alain Kohl

**Affiliations:** 1 MRC-University of Glasgow Centre for Virus Research, Glasgow, Scotland, United Kingdom; 2 Fundação Oswaldo Cruz-PE/Centro de Pesquisas Aggeu Magalhães, Departamento de Virologia, Campus da UFPE-Cidade Universitária, Recife/PE, Brasil; 3 Faculty of Science, University of South Bohemia, České Budějovice, Czech Republic; 4 Institute of Parasitology, Biology Centre of the Academy of Sciences of the Czech Republic, České Budějovice, Czech Republic; 5 Department of Microbial Pathogenesis, Yale University, New Haven, Connecticut, United States of America; 6 Pole de Virologie, Unité des arbovirus et virus des fièvres hémorragiques, Institut Pasteur de Dakar, Dakar, Senegal; 7 Boyd Orr Centre for Population and Ecosystem Health, Institute of Biodiversity, Animal Health and Comparative Medicine, College of Medical Veterinary and Life Sciences, University of Glasgow, Glasgow, Scotland, United Kingdom; 8 Medical Research Council Human Immunology Unit, Weatherall Institute of Molecular Medicine and Radcliffe Department of Medicine, University of Oxford, Oxford, England, United Kingdom; University of California, Davis, UNITED STATES

## Abstract

**Background:**

The outbreak of Zika virus (ZIKV) in the Americas has transformed a previously obscure mosquito-transmitted arbovirus of the *Flaviviridae* family into a major public health concern. Little is currently known about the evolution and biology of ZIKV and the factors that contribute to the associated pathogenesis. Determining genomic sequences of clinical viral isolates and characterization of elements within these are an important prerequisite to advance our understanding of viral replicative processes and virus-host interactions.

**Methodology/Principal findings:**

We obtained a ZIKV isolate from a patient who presented with classical ZIKV-associated symptoms, and used high throughput sequencing and other molecular biology approaches to determine its full genome sequence, including non-coding regions. Genome regions were characterized and compared to the sequences of other isolates where available. Furthermore, we identified a subgenomic flavivirus RNA (sfRNA) in ZIKV-infected cells that has antagonist activity against RIG-I induced type I interferon induction, with a lesser effect on MDA-5 mediated action.

**Conclusions/Significance:**

The full-length genome sequence including non-coding regions of a South American ZIKV isolate from a patient with classical symptoms will support efforts to develop genetic tools for this virus. Detection of sfRNA that counteracts interferon responses is likely to be important for further understanding of pathogenesis and virus-host interactions.

## Author Summary

The current ZIKV outbreak is a major public health concern in the Americas. To further understand the virus, and to develop tools and potentially vaccines, more information on the virus strains circulating in the Americas is required. Here we describe the full-length sequence of a ZIKV isolate from a patient with classical symptoms, including the complete non-coding regions which are missing from many currently available sequences, and put these in context. Moreover, we also demonstrate the production of an RNA molecule derived from the 3’ untranslated region that counteracts interferon responses and may therefore be important for understanding the pathogenesis of ZIKV infection.

## Introduction

Zika virus (ZIKV) is a mosquito-transmitted arbovirus in the *Flavivirus* genus, *Flaviviridae* family. This previously obscure virus has recently caused large scale outbreaks in French Polynesia in 2013 [1, 2], New Caledonia [3], the Cook Islands [4] and Easter Island [5] in 2014 and the Americas in May 2015, beginning in Brazil [6, 7]. These outbreaks have been characterized by an increased prevalence of neurological syndromes, such as Guillain-Barré syndrome and microcephaly [8–13], which has heightened public concern. As of April 2016 the World Health Organization (WHO) announced that 60 countries had reported autochthonous transmission in the escalating epidemic originating in Bahia, Brazil in 2015 that has so far resulted in over 1.5 million suspected cases [14]. This unprecedented spread combined with the associated neurological conditions resulted in WHO declaring a global public health emergency in February 2016.

Brazil has the greatest burden of dengue virus (DENV), a related flavivirus, in the world and the ongoing ZIKV epidemic is occurring in areas where such mosquito-borne arboviruses are a major public health problem. This is due to widespread arbovirus vectors such as *Aedes aegypti* and *Ae*. *albopictus* which are important vectors of DENV and chikungunya virus (CHIKV, *Togaviridae*), as well as ZIKV [15–19]. Clinical manifestations of ZIKV are similar to symptoms of DENV or CHIKV infections making misdiagnosis common [3, 20]. Only 20% of ZIKV infections are thought to progress to clinical symptoms, which present as an acute, self-limiting illness comprising fever, myalgia, headache, polyarthralgia, nonpurulent conjunctivitis and maculopapular rash. The largest public health risk from ZIKV is its association with neurological conditions such as Guillain-Barré syndrome and microcephaly which place substantial strains on local communities and healthcare providers.

As is characteristic of flaviviruses, ZIKV possesses a linear single-stranded, positive-sense RNA genome. The flavivirus genome has a single open reading frame that encodes all structural and non-structural proteins flanked by 5´ and 3´ untranslated regions (UTRs) [21]. Phylogenetic analysis of partial ZIKV sequence data revealed isolates may be categorised into African and Asian lineages, of which the African lineage is further subdivided into Nigerian and MR766 prototype strain clades [22, 23]. Recently obtained sequences from the current epidemic are of Asian lineage and are most closely related to strains from the French Polynesian outbreak in 2013 [5, 6, 24]. However, there are currently few full-length complete sequences that include the genome termini. One of these is from the Americas and was derived from a microcephaly case [10]. Nonetheless, such information is important given the relevance of the genome termini and non-coding regions in virus translation, replication and pathogenesis. The 5’ and 3’ non-translated regions of flavivirus genomes have been shown to demonstrate conserved secondary structures, cyclization elements, and are important for binding to several host proteins in addition to proteins involved in viral replication complexes [25, 26]. Furthermore, the 3’UTR encodes subgenomic flavivirus RNA (sfRNA) which is produced by the incomplete degradation of viral RNA by a cellular 5’-3’ exoribonuclease [27, 28]. These molecules have been shown to be more than a by-product and are involved in viral interference with innate immune responses in both vertebrates and invertebrates through antagonizing type I interferon and RNA interference responses respectively [29–36].

Herein we present the complete genome sequence of a ZIKV isolate derived from a patient in Brazil with classical disease symptoms. This will be important for future studies and the development of reagents, such as reverse genetics systems, for ZIKV. We also identified ZIKV-derived sfRNA in infected cells and show that it functions as an antagonist of RIG-I mediated induction of type I interferon, while a lesser effect on MDA-5 mediated induction was observed. The production of sfRNA in ZIKV infection may be an important contributor to associated pathogenesis.

## Materials and Methods

### Ethics statement

This study was approved by the Brazilian Ethics Committee, Process number: IMIP Human Ethics Research Committee Approval number 4232, PlatBr580.333 and 44462915.8.2004.5190. The virus reported here, *ZIKV/H*. *sapiens/Brazil/PE243/2015* (abbreviated to ZIKV PE243), was isolated in Recife (Brazil) in 2015 from a patient (rash on face and limbs; arthralgia hands, fist/wrist, ankle; edema on hands, fist/wrist; no neurological symptoms). All patients who agreed to participate in this study were asked to sign an informed consent form.

### Virus isolation from cell culture

ZIKV from positive serum samples was isolated at Fundação Oswaldo Cruz (FIOCRUZ), Recife (Brazil) by amplification in C6/36 *Ae*. *albopictus* cells. then Vero cells, which are frequently used for virus isolation and were obtained from collections at FIOCRUZ. Briefly, 50 μl of positive serum was incubated for 1 h at room temperature on monolayers of C6/36 cells. The cells were then further incubated for 7 days. Following this, ZIKV infection was confirmed by RT-PCR as described below.

### Viral RNA extraction and RT-PCR

Viral RNA was extracted from serum of suspected acute DENV/ZIKV cases using the QIAmp Viral RNA Mini kit (Qiagen) following the manufacturer’s instructions. RNA was extracted from 140 μl of the sample and stored at -70°C prior to downstream applications. RT-PCR was carried out using the QIAGEN OneStep RT-PCR kit in a final volume of 25 μl following previously established protocols and primers [22].

### Virus growth and titration by plaque assay

Vero E6 cells, a commonly used cell line for the growth of viruses [37] were infected with ZIKV PE243 for the preparation of virus stocks which were collected upon detection of cytopathic effect. ZIKV PE243 infected cells tested positive with mouse anti-ZIKV serum (provided by G. Fall and A. A. Sall, Institut Pasteur de Dakar, Senegal) as well as with commercially obtained ZIKV E protein-specific antibodies by western blotting and immunofluorescence (S1 File). For titration, Vero E6 cells were infected with serial dilutions of virus and incubated under an overlay consisting of DMEM supplemented with 2% FCS and 0.6% Avicel (FMC BioPolymer) at 37°C for 5–7 days. Cell monolayers were fixed with 4% formaldehyde. Following fixation, cell monolayers were stained with Giemsa to visualize plaques. Plaque assays for plaque size comparisons were also performed using A549 and A549/BVDV-Npro cell lines (provided by R. E. Randall, University of St Andrews, UK) [37–39].

### Detection of ZIKV PE243 sfRNA by northern blot

Denaturated total RNA (3.5 μg per sample; isolated from Vero E6 cells infected with ZIKV PE243 at an multiplicity of infection [MOI] of 1 by Trizol followed by Direct-zol RNA purification) was separated on a denaturating formaldehyde agarose gel (1.5% agarose, 1x MOPS buffer [Fisher Scientific], 12.3 M formaldehyde) in 1x MOPS running buffer. RNA was transferred onto a Hybond-N+ membrane (GE Healthcare Life Sciences) via capillary transfer action using 10x SSC (1.5 M NaCl, 150 mM trisodium citrate). RNA was crosslinked to the membrane by UV (120 mJ/cm^2^). Following transfer, the membrane was prehybridized for 2 h in PerfectHyb Plus Hybridization buffer (Sigma-Aldrich) at 65°C. Specific oligonucleotides for the sfRNA region of the ZIKV PE243 3’UTR (forward: AGCTGGGAAACCAAGCCTAT, reverse: GTGGTGGAAACTCATGGAGTCT) were used to amplify a fragment by PCR with KOD polymerase (Merck Millipore). Following this 250 ng of the PCR product was end-labelled with ^32^P using T4 Polynucleotide Kinase (NEB) and [γ-^32^P]Adenosine 5’-triphosphate (PerkinElmer) to produce a probe. The probe was denatured for 5 min at 95°C and added to prehybridization mixture which was incubated on the membrane overnight at 65°C. The membrane was then washed twice for 15 min at 65°C with each of the following three buffers: 2x SSC and 0.5% SDS, 2x SSC and 0.2% SDS, 0.2x SSC and 0.1% SDS. RNA species were detected by phosphorimaging.

### Cloning of ZIKV 3’UTR

The Gateway cloning system was used for cloning the 3’UTR of ZIKV, potentially containing the sfRNA sequence, fused to hepatitis delta virus ribozyme (HDVr) into pDEST40 (mammalian expression vector [Invitrogen]). The 3’UTR of ZIKV PE243 was amplified by PCR using 1 μl of the 3' end RACE reaction as a template. Subsequently, fusion PCR was performed using the primers described in Table 1. The resulting fragment was inserted into the pDONR207 using BP Clonase II kit (Invitrogen) and sequenced using the pDONR201 forward primer. LR Clonase II kit (Invitrogen) was used for the recombination of pDONR207-ZIKV PE243-3’UTR (entry vector) and the empty pDEST40 resulting in pDEST40-ZIKV PE243-3’UTR. The sequence of pDEST40-ZIKV PE243-3’UTR was validated using the T7 promoter forward primer. Similar cloning strategies have been used for other flavivirus 3’UTRs containing sfRNA [29, 30].

**Table 1:** Primers used for cloning of ZIKV 3'UTR containing sfRNA.

Primer	Use	Sequence (5'-3')
ZIKV-3’UTR-FW	ZIKV 3'UTR amplification	GCACCAATCTTAATGTTGTCAGG
ZIKV-3’UTR-RV		AGACCCATGGATTTCCCC
ZIKV-3’UTR-attB-FW	amplification of attB-ZIKV 3'UTR-HDVr fragment	GGGGACAAGTTTGTACAAAAAAGCAGGCTTCGCACCAATCTTAATGTTGTC
ZIKV-3’UTR-HDVR-RV		CATGCCGACCCAGACCCATGGATTTCCCCA
HDVR-ZIKV-3’UTR-FW	amplification of ZIKV 3'UTR-HDVr-attB fragment	GAAATCCATGGGTCTGGGTCGGCATGGCATCTC
HDVR-attB-RV (E10)		GGGGACCACTTTGTACAAGAAAGCTGGGTTTTCCGATAGAGAATCGAGAGAAAA
pDONR201 forward	sequencing (pDONR207)	TCGCGTTAACGCTAGCATGGATCTC
T7 promoter (F)	sequencing (pDEST40)	TAATACGACTCACTATAGGG

### Interferon assays

*In vitro* type I interferon assays were performed using the human A549 cell line [37] to analyze the activity of the IFN-β promoter in the presence of plasmids expressing flavivirus 3’UTRs containing the sfRNA sequence. A549 cells were grown in DMEM (supplemented with 10% FBS, 1000 units/ml penicillin and 1 mg/ml streptomycin) at 37°C with 5% CO_2_. Briefly, 24 h prior to transfection, A549 cells were seeded in 24 well plates at a density of 1.2x10^5^ cells/well to reach 70% confluency the following day. Cells were first co-transfected with 400 ng p125Luc IFN-β promoter reporter vector expressing Firefly luciferase [40], 2 ng pRL-CMV (internal control, expressing *Renilla* luciferase), and 500 ng of either pDEST40 expressing DENV [29] or ZIKV 3’UTRs (constructs described in this study) or a MBP-HDVr (maltose-binding protein-HDVr) control using Opti-MEM and Lipofectamine2000 (Invitrogen) according to the manufacturer’s protocol. Following a further 24 h incubation, type I interferon induction was stimulated by transfecting the cells a second time with either 10 μg/well poly I:C, 50 ng Vero cell produced EMCV RNA or 50 ng Neo^1-99^ IVT-RNA (universal, MDA-5 specific and RIG-I specific type I interferon agonists respectively) [41, 42]. Cells were lysed in 1x passive lysis buffer (Promega) 24 h after the second transfection and Firefly and *Renilla* luciferase activities determined using a Dual-Luciferase reporter assay kit (Promega) in a GloMax luminometer.

### Virus infection for RNA sequencing

Vero E6 cells were infected with ZIKV at an MOI of 0.001 in triplicate. At 48 h post infection (p.i.), cell culture supernatant was harvested and clarified by low speed centrifugation. Following clarification, 6 ml of infected cell supernatant was concentrated to 250 μl using an Ultra-15 Centrifugal Filter Units with 100 kDa molecular weight cut-off (Amicon). Concentrated supernatant was then added to Direct-zol solution and RNA extracted using a Direct-zol RNA mini kit (Zymogen) according to the manufacturer’s instructions. Purified RNA was then stored at -80°C for further downstream processing.

### RACE analysis of viral genome termini

Sequencing of the 5’ and 3’ termini of the viral genome was performed using a 5’/3’ RACE kit (Roche) following the manufacturer’s protocol. All primers used are described in Table 2. To obtain the 5’ end sequence of the ZIKV genome 5’ RACE was performed. Briefly, 1 μg total RNA was extracted from ZIKV-infected Vero E6 cells using a Direct-zol RNA mini kit and reverse transcribed using the ZIKV specific primer, SP1. The synthesized cDNA was purified using the illustra GFX PCR DNA and Gel Band Purification kit (GE Healthcare) according to the manufacturer’s instructions. This was prior to polyadenylation at the 3’ end and amplification using the PCR anchor primer and a ZIKV specific primer (5’ PCR). 3’ RACE was carried out to obtain the 3’ end sequence using 1 μg total RNA extracted from ZIKV infected Vero cells which was polyadenylated at the 3’ end using Poly(A) polymerase (New England Biolabs) following the manufacturer’s guidelines. cDNA synthesis was performed by reverse transcribing the RNA using the oligo (dT) anchor primer. Amplification of the cDNA was achieved by using the PCR anchor primer and a ZIKV specific primer (3’ PCR). The PCR cycling conditions were 95°C for 2 min then 35 cycles of 95°C 20 sec, 56°C (5’ RACE) or 68°C (3’ RACE) for 10 sec, 70°C for 15 sec and 70°C for 7 min.

**Table 2:** Primers sequences used for 5’/3’ RACE of ZIKV viral termini.

Primer	Use	Sequence (5’-3’)
SP1	cDNA synthesis	CTCATGGTGGCATCACACATGTGTCCAAGATCC
5’ PCR	PCR amplificiation	TGCACTCCCACGTCTAG
3’ PCR	PCR amplification	TGGCCAATGCCATTTGTTCATCTGTGC
5’ SEQ	Sequencing	CATCTATTGATGAGACCCAGTGATGGC
3’ SEQ	Sequencing	GAAGACTTGTGGTGTGGATCTCTCATAGGGCACAG
3’ SEQ2	Sequencing	GCCTGAACTGGAGATCAGCTGTGGATC

### cDNA synthesis and NGS library preparation

A volume of 25 μl of cell culture supernatant was treated with RNase-free DNase I (Ambion), purified with RNAClean XP magnetic beads (Beckman Coulter) and eluted in 11 μl of water. In parallel, an equivalent sample was concentrated from 25 to 11 μl using magnetic beads as indicated above, in the absence of DNase I treatment. In addition, 45 μl of extracted total cellular nucleic acid was treated with RNase-free DNase I and purified as above. Half of the volume was further depleted of ribosomal RNA (RiboZero Gold) according to the manufacturer's protocol.

All samples were reverse-transcribed using Superscript III (Invitrogen) followed by dsDNA synthesis with NEB Next(r) mRNA Second Strand Synthesis Module (New England Biolabs). Libraries were prepared using a KAPA DNA Library Preparation Kit (KAPA Biosystems), utilizing a modified protocol that includes ligation of the NEBnext adapter for Illumina (New England Biolabs), followed by indexing with TruGrade oligonucleotides (Integrated DNA Technologies) to eliminate tag crossover. Resulting libraries were quantified using a Qubit 3.0 fluorometer (Invitrogen) and their size determined using a 2200 TapeStation (Agilent). Libraries were pooled in equimolar concentrations.

### Sequencing analysis

Samples from different passages were sequenced on a NextSeq500 platform (Ilumina). This obtained 24,275,098 read pairs (2x150bp) and 88.8% of reads had a quality score of >Q30.

### Bioinformatic analysis

Reads were first checked for quality using FASTQC (http://www.bioinformatics.babraham.ac.uk/projects/fastqc/) and trimmed for adapter sequences and quality filtered using trim_galore (http://www.bioinformatics.babraham.ac.uk/projects/trim_galore/). These were subsequently mapped to the ZIKV complete genome KU321639 using two different aligners: Tanoti (http://www.bioinformatics.cvr.ac.uk/tanoti.php) and Bowtie2 [43]. The assembly was parsed using customized scripts to determine the frequency of nucleotides at each site and reconstruct a consensus with nucleotides above 50%. The complete genome was extended at the 5’ and 3’UTRs by extracting additional reads that overlapped with the terminal ends of the consensus sequence. The sequence of the ZIKV PE243 genome has been deposited in GenBank with the accession number KX197192.

### Phylogenetic and sequence analysis

Phylogenetic and comparison analyses were carried out using full coding sequence alignments that were generated using MUSCLE [44] within the program suite Geneious (version 7.1.8: http://www.geneious.com) [45]. These alignments were created using our ZIKV PE243 sequence in addition to publicly available coding sequences on GenBank. All Asian and African lineage ZIKV sequences used for the analysis are described in S1 Table. A single African sequence (MR-766, accession NC_012532) was used as an outgroup. Before generating phylogenies, the data set was analyzed for the presence of recombination. The Recombination Detection Program version 4 (RDP4) [46] software was utilized, specifically the programs RDP, Chimaera, BootScan, 3Seq, GENECONV, MacChi & SiScan. Phylogenies were generated with both maximum likelihood and Bayesian inference methods using the software packages PhyML [47] and MrBayes (version 3.2.6) [48] respectively. Support for the maximum likelihood tree topology was generated by 1,000 non-parametric bootstrap replicates. For the Bayesian analysis one MCMC run of four heated chains of length 1,000,000 was utilized to ensure an effective sample size of at least 200. The run was sampled every 200^th^ generation and the first 10% of samples were discarded as burn-in. The generalized time reversible (GTR) substitution model with gamma distribution (+G) was found to suit the data set best, as selected by both jModel Test [49] and HyPhy [50] software packages. The topologies of both the Bayesian and maximum likelihood trees were identical; here we present only the Bayesian tree.

### Statistical analysis

All data were analysed using Prism 5 software (GraphPad) and presented as mean ± standard error. Statistical significance for the comparison of means between groups was determined by a two-way ANOVA; p values ≤0.05 were considered significant.

## Results and Discussion

### Characterization of *ZIKV/H*. *sapiens/Brazil/PE243/2015*

At the time of writing, 62 ZIKV genomes are available on GenBank, of which 37 are published. Of these only 11 showed both 5’ and 3’ complete UTRs (accessed 16th April 2016). A summary of currently available strain information and accession numbers is presented in S1 Table. ZIKV PE243 was isolated from a patient presenting with classical symptoms associated with ZIKV infection and the complete viral genome sequence including the non-coding regions was determined. The UTRs are largely missing in many sequences from the Americas, with some exceptions including the Natal isolate derived from a case presenting with microcephaly [10]. Only recently have more full-length ZIKV sequences been described [51, 52].

**Fig 1:**
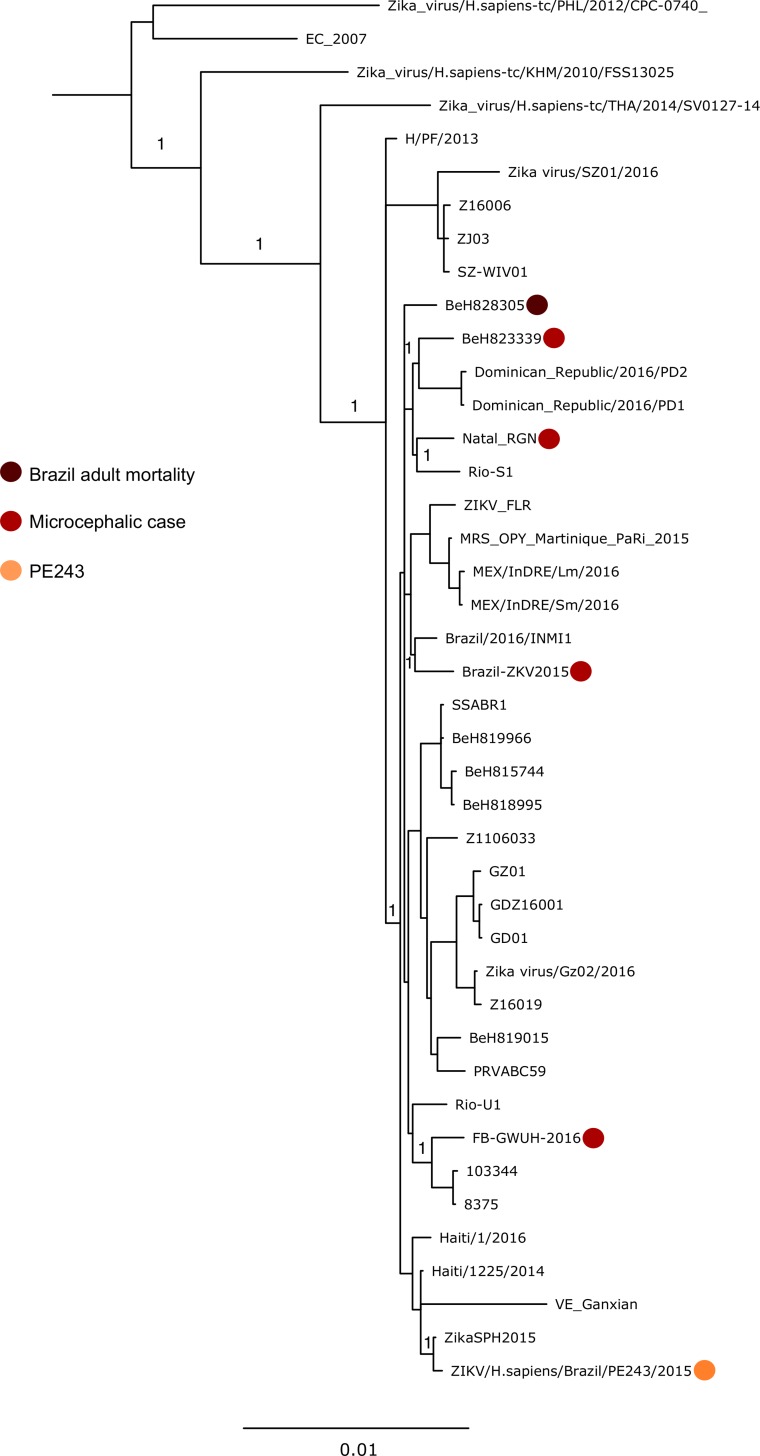
Bayesian maximum clade credibility tree generated from coding sequence data. Bayesian posterior probabilities are given at nodes of importance. Isolates which have been implicated in particular diseases are highlighted, as is the ZIKV PE243 isolate we have sequenced. GenBank accession numbers of all sequences used are given in S1 Table. EC_2007 refers to the epidemic consensus sequence generated from the Yap Island outbreak in 2007 (EU545988).

**Fig 2:**
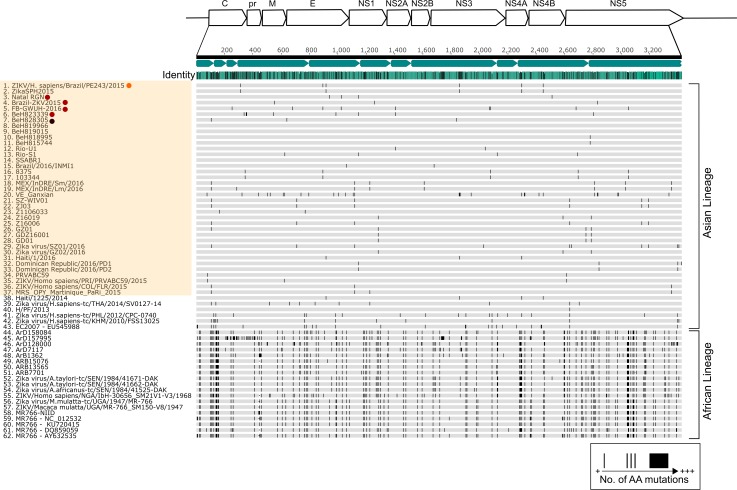
Comparison of African and Asian lineage ZIKV protein coding regions. The mean pairwise identity of all pairs at a given position is indicated by the identity bar; light blue denotes 100% pairwise identity, dark blue highlights positions possessing less than 100% pairwise identity. Positions and quantity of amino acid substitutions are indicated by black bands within grey sequence bars. Sequences 1–37, highlighted yellow, correspond to the outbreak originating in 2015 in South America. Microcephaly, adult mortality and ZIKV PE243 associated sequences are highlighted as previously described in Fig 1.

Our phylogenetic analysis uses the entire protein-coding region and the position of our isolate was supported by a posterior probability node support of 1. Recombination screening prior to analysis also produced no signals. The sequence of ZIKV PE243 used for further analysis (as deposited in GenBank) derives from virus that had been passaged five times in Vero E6 cells upon receipt by the Centre for Virus Research (Glasgow, UK) on a NextSeq500 (average depth of coverage of 5637, range 52–13691). Three nucleotide substitutions were observed following the sequencing of this virus compared to a previous passage of the isolate (passage two) that had been sequenced on a MiSeq platform (these earlier data did not generate complete coverage; average depth of coverage of 1158, range 2–2944). The mutations observed are as follows: site 2784, 1149 out of 1159 reads had A in the MiSeq run (after two passages) and 3508 out of 3910 reads had G in the NextSeq run after a further three passages. The mutation A2784G corresponds to the amino acid substitution R893G in NS1. The mutations observed in NS3 (U5231C: 1727/1730 Ts in passage two versus 7031/7623 Cs in passage five) and NS4B (A7637G: 1835/1846 As in passage two versus 9578/10587 Gs in passage five) were synonymous. These three substitutions represent mutations obtained during adaptation in cell culture between passage two and passage five. The mutations A2784G and U5231C are unique to ZIKV PE243 and are not found in any other strains published to date. Phylogenetic analysis based on the entire protein coding region grouped the ZIKV PE243 isolate with another 2015 Brazilian isolate (KU321639, ‘ZikaSPH2015’) with 100% posterior support (Fig 1). As expected, our isolate clusters with other strains from the Americas which belong to the Asian lineage that is attributed to the epidemic in French Polynesia in 2013 (Fig 1). Previous findings have shown that American isolates are genetically very comparable, with approximately 99% homology at the nucleotide level, and there is less than 12% diversity between strains from both African and Asian lineages [24, 53]. Our data are in agreement with this as ZIKV PE243 demonstrates a strong degree of conservation at amino acid level (98.3% pairwise identity) with sequences from 62 isolates (Fig 2). ZIKV PE243 shares the greatest level of similarity with the Brazilian isolate ZikaSPH2015 (99.9% at the nucleotide level and 99.97% at amino acid level) [54] and the passage two isolate matched the coding region precisely. There is no obvious virological explanation, based upon our sequence analysis, for the increased occurrence of neurological disease cases associated with the outbreak in Brazil. This is in accordance with other findings which have similarly suggested that there are no specific mutations in the viral genome associated with severe cases [54]. However, the role of mutations in ZIKV isolates needs to be assessed by reverse genetics approaches to provide conclusive evidence.

**Fig 3:**
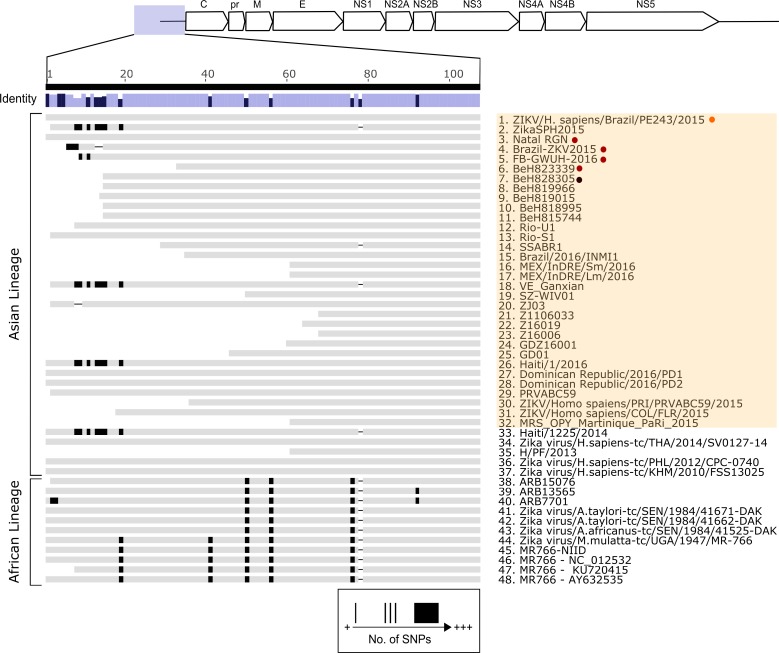
Comparison of the 5’UTR nucleotide sequences of Asian and African ZIKV isolates. The mean pairwise identity of all pairs at a given position is indicated by the identity bar; lilac is indicative of 100% pairwise identity, dark purple highlights positions possessing <100% pairwise identity. Positions and quantity of single nucleotide polymorphisms (SNPs) are represented as black bands within grey sequence bars. Sequences 1–32, highlighted orange, correspond to the outbreak originating in 2015 in Brazil. Microcephaly, adult mortality and ZIKV PE243 associated sequences are highlighted as previously described in Fig 1.

**Fig 4:**
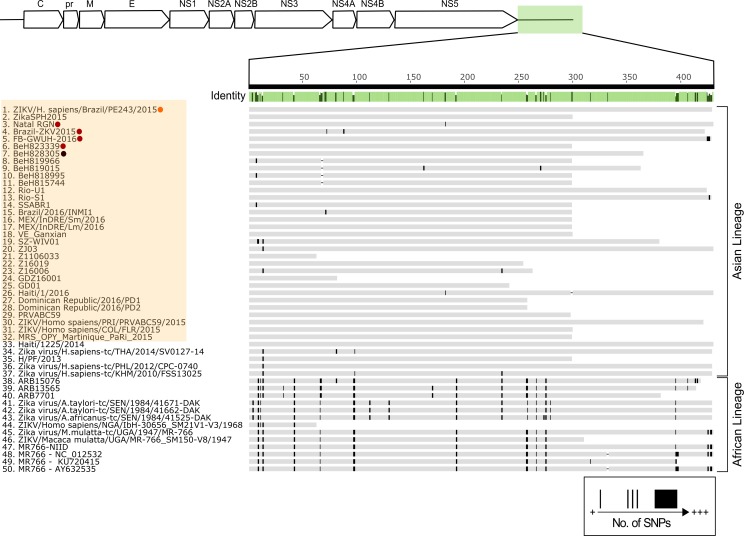
Comparison of the 3’UTR nucleotide sequences of Asian and African ZIKV isolates. The mean pairwise identity of all pairs at a given position is indicated by the identity bar; light green is indicative of 100% pairwise identity, dark green highlights positions possessing less than 100% pairwise identity. Sequences 1–32, highlighted orange, correspond to the outbreak originating in 2015 in Brazil. Microcephaly, adult mortality and ZIKV PE243 associated sequences are highlighted as previously described in Fig 1.

We also successfully sequenced both the 5’ and 3’ non-coding regions (Figs 3 and 4). Of the 62 sequences publicly available (as of 16^th^ April 2016), 48 sequences with 5’UTR information are shown in the consensus alignment (Fig 3). ZIKV strains ZIKV/*Homo sapiens*/NGA/ibH-30656_SM21V1-V3/1968 and ZIKV/*Macaca mulatta*/UGA/MR-766_SM150-V8/1947 contain large insertions and were subsequently excluded from 5’UTR analysis. The 5’UTR of ZIKV PE243 shares 100% sequence identity with the consensus sequence (the most common bases between all sequences analyzed) and overall very few mismatches are detected across all 48 sequences studied. The 5’UTR is largely conserved between isolates of the same lineage and is approximately 107 nucleotides long in isolates from the Asian lineage, similar to the length shown for MR766 strain and other African lineage viruses. There was strong similarity between ZIKV PE243 and Natal RGN, a Brazilian isolate associated with microcephaly [10], while ZIKV PE243 was associated with classical symptoms. Similarly, there are few mismatches between known 3’UTRs (Fig 4). These non-coding regions are expected to be approximately 428 nucleotides in length as seen for many Asian and African isolates.

### ZIKV PE243 produces interferon antagonist sfRNA

The host interferon response is known to be essential for fighting viral infections and preventing virus replication, including mosquito-borne flaviviruses [55–58]. This has been specifically illustrated for ZIKV as *in vivo* pathogenesis studies require murine models lacking type I interferon [59], while type III interferon has been shown to have a protective role against ZIKV infection in human placental cells [60]. Furthermore, ZIKV NS5 has recently been described as a type I IFN signaling antagonist that targets STAT2 [61]. Indeed, ZIKV PE243 was also susceptible to type I interferon responses and produced much larger plaque sizes in the type I interferon incompetent A549/BVDV-Npro cell line than in A549 cells (S1 Fig).

**Fig 5:**
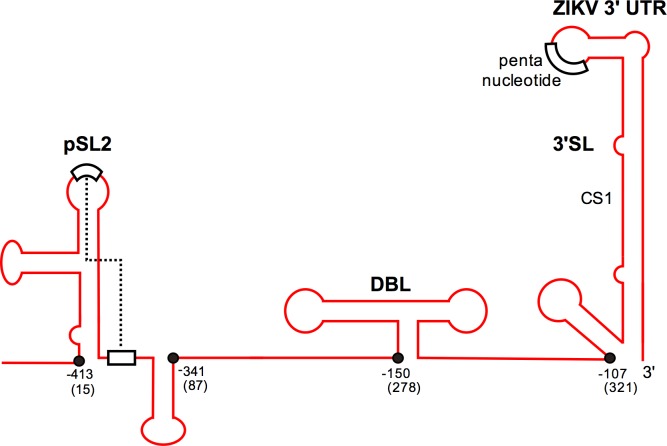
The predicted structure of ZIKV PE243 3’UTR. 5’-3’ of the ZIKV PE243 3’UTR sequence, left to right. The arrow indicates the predicted start of sfRNA. Nucleotides are indicated either counted from the 3’ (indicated as negative numbers) or from the start of the 3’UTR (positive number in brackets). SL, stem loop structure; DBL, dumbbell structure; 3’SL; 3’ end stem loop structure. The dotted line represents the predicted pseudoknot.

However, viruses also employ mechanisms that allow them to counteract the host’s interferon responses in order to replicate efficiently. Mosquito- and tick-borne flaviviruses express sfRNA derived from the 3’ terminus, which is resistant to RNase (XRN1)-mediated virus genome degradation due to RNA stem loop structures and pseudoknots in this region [27, 28]. Interestingly, sfRNA has been implicated in pathogenesis, immune evasion and inhibition of small RNA-based responses [29–34]. Thus, a similar subgenomic RNA produced during ZIKV infection could be important in the development of disease and virus-host interactions. Based on our sequence data and comparisons to other mosquito-borne flavivirus 3’UTRs, we predicted the structure of ZIKV PE243 sfRNA (Fig 5). Secondary structures, specific for flavivirus 3’UTRs, were detected in the 3’UTR of ZIKV PE243 by Clustal alignments of the 3’UTR of ZIKV PE243, yellow fever virus (X03700, K02749), DENV2 (M19197), Kunjin virus (AY274504), Japanese encephalitis virus (AF014161) and Murray Valley encephalitis virus (AF161266) in combination with Mfold. Putative pseudoknot interactions were determined by hand. Further analysis was also carried out to compare the 3’UTR sequences between ZIKV PE243 and 3 African strain isolates (two MR766 isolates [AY632535, KX377335] and another African isolate [KU955592]). Our comparisons suggest that the sequence differences between these Asian and African isolates do not, or are unlikely to, affect the predicted sfRNA structure (S2 Fig, S3 Fig and S2 Table).

**Fig 6:**
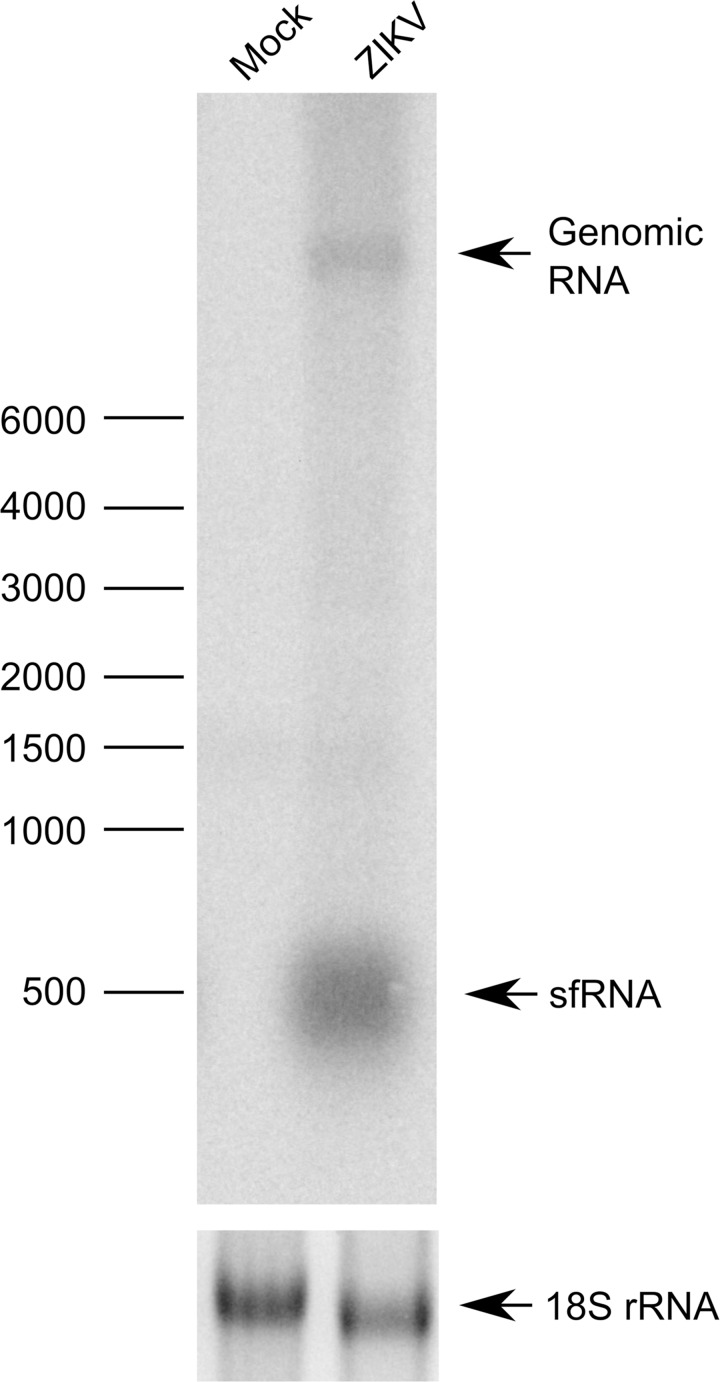
sfRNA production in ZIKV PE243 infection. Top panel: Vero E6 cells were infected with ZIKV isolate PE243 and sfRNA detected by northern blot. Total RNA isolated from Vero E6 cells infected with ZIKV PE243 was separated on a denaturing agarose gel and transferred to a nylon membrane as described in Materials and methods. Radiolabeled DNA probe complementary to 3’UTR was used to detect genomic RNA and sfRNA. Bottom panel: assessed amounts of 18S ribosomal RNAs (fluorescently labelled with ethidium bromide) prior to transfer.

**Fig 7:**
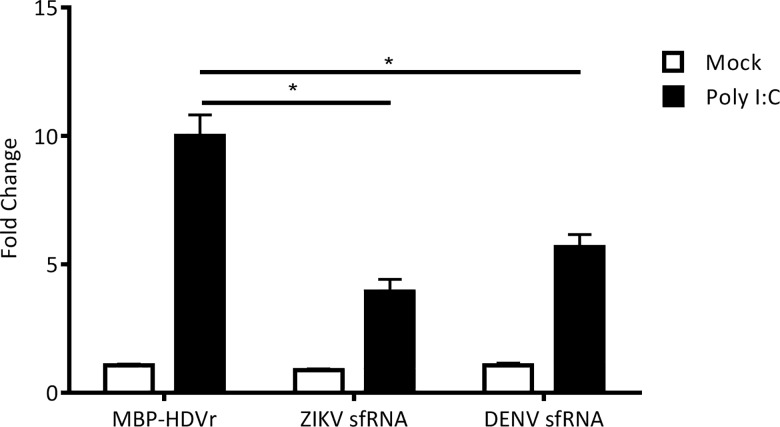
Activation of the IFN-β promoter by poly I:C in cells over expressing ZIKV sfRNA. A549 cells were co-transfected with either pDEST-DENV-3’UTR, pDEST-ZIKV PE243-3‘UTR or pDEST40-MBP (sfRNA over-expression plasmids and MBP-HDVr control, respectively) and p125Luc IFN-β promoter reporter (expressing Firefly luciferase) along with pRL-CMV (internal control, expressing *Renilla* luciferase). The IFN-β promoter was stimulated by transfecting poly I:C 24 h after the primary transfection. The relative luciferase activity (Firefly/*Renilla*) was analyzed at 24 h following the second transfection. The mean with standard error is shown for three independent experiments performed in triplicate; values of independent experiments were used for analysis. The data were normalized to cells transfected with pDEST40-MBP without any poly I:C treatment. Asterisk (*) indicates significance (2-way ANOVA, p<0.05).

Our sequence data for ZIKV PE243 and predictive analysis suggested that the ZIKV sfRNA molecule begins 15 nt after the stop codon of the open reading frame and is 413 nt in length. This was further confirmed by northern blot analysis, which indicates a band at the anticipated size present only in ZIKV PE243 infected cell lysate (Fig 6). It is important to determine whether this molecule is involved in inhibition of type I IFN production as previously described for other flavivirus sfRNAs [27]. To test this hypothesis, cells were co-transfected with a reporter plasmid (p125Luc) expressing Firefly luciferase under the control of the IFN-β promoter as well as plasmids expressing either ZIKV or DENV 3’UTRs which contain the sfRNA sequences. The IFN-β promoter was stimulated by treating with poly I:C (Fig 7).

As demonstrated in Fig 7, ZIKV PE243 sfRNA reduced activation of the IFN-β promoter to the same level as DENV sfRNA compared to MBP-HDVr control. This shows that ZIKV sfRNA functions in a similar manner to other flavivirus sfRNA molecules and interacts with important innate immune responses that may impact on virus replication and thus the severity of the clinical outcome.

**Fig 8:**
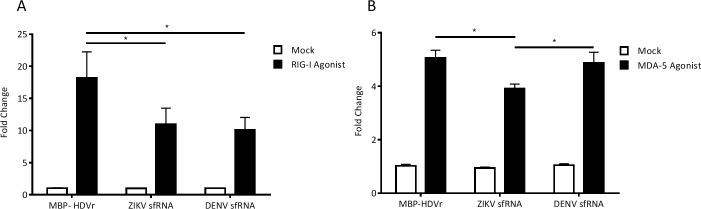
Activation of the IFN-β promoter by RIG-I or MDA-5 agonists in cells over-expressing ZIKV sfRNA. A549 cells were co-transfected as described with either pDEST-DENV-3’UTR, pDEST-ZIKV PE243-3‘UTR or pDEST40-MBP and p125Luc IFN-β promoter reporter along with pRL-CMV. The IFN-β promoter was stimulated by transfecting either RIG-I agonist (Neo^1-99^ IVT-RNA) (A) or MDA-5 agonist (Vero cell produced EMCV RNA) (B) 24 h after the primary transfection. The relative luciferase activity (Firefly/*Renilla*) was analyzed at 24 h following the second transfection. The mean with standard error is shown for three independent experiments performed in duplicate; values of independent experiments were used for analysis. The data were normalized to cells transfected with pDEST40-MBP without any agonist treatment. Asterisk (*) indicates significance (2-way ANOVA, p<0.05).

To further understand the mechanism of action ZIKV sfRNA molecules use to antagonize the interferon response, the above assay was repeated this time using specific inducers of type I interferon induction components, RIG-I and MDA-5 [41, 42]. Receptors such as RIG-I and MDA-5 signal for the induction of IFN-α/β production through the detection of viral nucleic acid [62, 63]. As shown in Fig 8, stimulation of RIG-I (Fig 8A) results in a significant decrease in IFN-β promoter activity in the presence of both DENV and ZIKV sfRNAs compared to the control. In contrast MDA-5 (Fig 8B) stimulation did not alter the activity of the IFN-β promoter in the presence of DENV sfRNA, although a weak but significant decrease was observed in ZIKV sfRNA expressing cells. These data suggest that both ZIKV and DENV antagonize RIG-I mediated type I interferon induction. Our data is consistent with previous findings for DENV sfRNA which found that DENV sfRNA binds TRIM25 interfering with its deubiquitylation, consequently hindering RIG-I mediated interferon induction [34]. Only ZIKV sfRNA antagonized MDA-5 activity in this assay, although the biological significance of this is yet to be clarified.

Over the past 40 years there has be an upsurge in the number of cases of important arbovirus infections such as DENV, CHIKV and West Nile virus (WNV), and ZIKV is now another emerging arbovirus of significant clinical importance. The factors involved in the emergence of ZIKV from a rarely detected pathogen to a major epidemic are yet to be determined and could include genetic adaptation, environmental influences, interactions with other pathogens within infected individuals and changes in population dynamics of the virus. To date, the northeast region of Brazil has reported a significant increase in cases of microcephaly and it is important to understand the determinants that lead to this clinical outcome. It has been suggested that alterations in codon usage in the NS1 gene may have facilitated an adaptation towards improved fitness for human infections in the Asian lineage over the African [64]. These changes, combined with the geographical ranges throughout the Americas of its vector population, may have contributed to its accelerated spread. More work is required to analyze these possibilities, and reverse genetics systems in particular will be key to studying mutations and genetic diversity within viral populations. The 5’ and 3’UTRs are important for virus replication and are therefore required for the development of such reverse genetic systems [65] that may be used in vaccine development or to advance knowledge of virus-host interactions. In order to understand not only ZIKV evolution and pathogenesis but also to support the development of virus-based tools, it is imperative to generate full virus genome sequences from ZIKV isolates in the Americas and elsewhere associated with classical and non-classical symptoms. Although new scientific information about ZIKV is published on a near daily basis, many avenues of research are yet to be fully explored in order to understand the clinical manifestations surrounding this outbreak. Characterization of the full sequence of ZIKV PE243 from a patient with symptoms classically associated with infection adds to our understanding of the virus genetics. We have also shown that ZIKV, like other pathogenic flaviviruses infecting humans, encodes sfRNA which inhibits type I interferon induction and thus is likely to contribute to viral pathogenesis. Our interferon induction assays suggest that ZIKV sfRNA may have broader antagonist activity compared to DENV sfRNA, which could contribute to disease outcome and requires further investigation. The data shown here give important insights into virus-host interactions that will help guide future research efforts in this field.

## Supporting Information

S1 Table ZIKV isolate information and data. (DOCX)

S2 Table Summary of 3’UTR mutations and associated secondary structures. Positions of single nucleotide mutations within the predicted sfRNA sequences of three African lineage isolates compared to ZIKV PE243 sfRNA. Mutations are described as Asian lineage: African lineage. * indicates MR766 conserved mutations.
(DOCX)

S1 Fig Type I interferon inhibits ZIKV PE243. Virus growth was analyzed by plaque size comparisons in human A549 (interferon competent) and A549/BVDV-Npro (type I interferon incompetent) cell lines.
(TIF)

S2 Fig Alignment and comparison between the 3’UTRs of ZIKV PE243 and 3 African lineage viruses. Accession numbers African ZIKV: MR766 isolates AY632535 and KX377335; further strain KU955592. Predicted sequence elements and structures are indicated.
(DOCX)

S3 Fig Location of African ZIKV 3’UTR mutations relative to ZIKV PE243. Shown is 5’-3’ of the ZIKV PE243 3’UTR sequence, left to right (as also shown in Fig 5). Asterisks indicate conserved mutations between all compared African lineage sequences (isolates AY632535, KX377335 and KU955592) and location in the predicted ZIKV PE243 sfRNA.
(TIF)

S1 File Data and methods for use of mouse anti-ZIKV serum as well as antibodies against ZIKV E protein. (DOCX)
