# Supplementary material for: Full Genome Sequence and sfRNA Interferon Antagonist Activity of Zika Virus from Recife, Brazil
Source: PLoS Negl Trop Dis. 2016 Oct 5;10(10):e0005048. doi: 10.1371/journal.pntd.0005048 (PMC5051680; doi:10.1371/journal.pntd.0005048)
Supplement: S1 Table — (DOCX) [file pntd.0005048.s001.docx]

| **Accession number** | **Strain/isolate** | **Location** | **Year of isolation** | **Host** | **Passage details** | **Genome length (nt)** | **5’ UTR length (nt)** | **3’ UTR length (nt)** |
| --- | --- | --- | --- | --- | --- | --- | --- | --- |
| KU527068.1*[[1](#_ENREF_1)] | Natal RGN | Brazil: Rio Grande do Norte, Natal | 2015 | *Homo sapiens* (foetus brain autopsy) | - | 10808 | 107 | 429 |
| LC002520.1 | MR766-NIID | Uganda | 1947 | Sentinel monkey | - | 10807 | 106 | 429 |
| KU955593.1*[[2](#_ENREF_2)] | Zika virus/H.sapiens-tc/KHM/2010/FSS13025 | Cambodia | 2010 | *Homo sapiens* | Vero (x1) | 10807 | 107 | 428 |
| KU681082.3*[[3](#_ENREF_3)] | Zika virus/H.sapiens-tc/PHL/2012/CPC-0740 | Philippines | 2012 | *Homo sapiens* | *Toxorhynchites splendens* mosquito (x1) followed by C6/36 (x1) | 10807 | 107 | 428 |
| KU681081.3*[[3](#_ENREF_3)] | Zika virus/H.sapiens-tc/THA/2014/SV0127- 14 | Thailand | 2014 | *Homo sapiens* | *Toxorhynchites splendens* mosquito (x1) followed by C6/36 (x1) | 10807 | 107 | 428 |
| KX197192.1* | ZIKV/H.sapiens/Brazil/PE243/2015 | Brazil | 2015 | *Homo sapiens* | Vero (x5) | 10807 | 107 | 428 |
| KX051563.1* | Haiti/1/2016 | USA | 2016 | *Homo sapiens* (saliva and urine) | LLC-MK2 cells (x1) | 10807 | 107 | 428 |
| KU509998.3** [[4](#_ENREF_4)] | Haiti/1225/2014 | Haiti | 2014 | *Homo sapiens* | - | 10807 | 106 | 429 |
| KU955595 [[2](#_ENREF_2)] | Zika virus/A.taylori-tc/SEN/1984/41671-DAK | Senegal | 1984 | *Aedes taylori* | AP61 cells (x1), C6/36 (x1), Vero (x3) | 10806 | 106 | 428 |
| KU955592.1 [[2](#_ENREF_2)] | Zika virus/A.taylori-tc/SEN/1984/41662-DAK | Senegal | 1984 | *Aedes taylori* | AP61 cells (x1), C6/36 (x1), Vero (x3) | 10806 | 106 | 428 |
| KU955591.1 [[2](#_ENREF_2)] | Zika virus/A.africanus-tc/SEN/1984/41525-DAK | Senegal | 1984 | *Aedes africanus* | AP61 cells (x1), C6/36 (x1), Vero (x3) | 10806 | 106 | 428 |
| KU926310.1* | Rio-S1 | Brazil | 2016 | *Homo sapiens* (saliva) | Vero | 10805 | 106 | 426 |
| KU820899.2* | ZJ03 | China | 2016 | *Homo sapiens* | - | 10805 | 104 | 429 |
| KU820897.2** | ZIKV_FLR | Colombia | 2015 | *Homo sapiens* | C6/36 (x1) | 10800 | 107 | 421 |
| KU870645.1* [[5](#_ENREF_5)] | FB-GWUH-2016 | Guatemala (putative country of infection) – fetal brain | 2016 | *Homo sapiens* (foetal brain) | SK-N-SH | 10798 | 99 | 427 |
| KU955594 .1 [[2](#_ENREF_2)] | Zika virus/M.mulatta-tc/UGA/1947/MR-766 | Uganda | 1947 | *Macaca mulatta* | Suckling mouse (x150), Vero (x2) | 10795 | 106 | 429 |
| KU926309.1* | Rio-U1 | Brazil | 2016 | *Homo sapiens* (urine) | Vero | 10795 | 99 | 422 |
| NC_012532 [[6](#_ENREF_6)] | MR766 | Uganda | 1947 | *Macaca mulatta* | - | 10794 | 106 | 398 |
| AY632535 [[7](#_ENREF_7)] | MR766 | Uganda | 1947 | *Sentinel monkey* | - | 10794 | 106 | 428 |
| KU497555.1* [[8](#_ENREF_8)] | Brazil-ZKV2015 | Brazil | 2015 | *Homo sapiens (amniotic fluid)* | - | 10793 | 100 | 421 |
| KF268948.1 [[9](#_ENREF_9)] | ARB13565 | Central African Republic | 1979 | *Aedes africanus* | - | 10788 | 107 | 413 |
| KF268949.1 [[9](#_ENREF_9)] | ARB15076 | Central African Republic | 1980 | *Aedes opok* | - | 10776 | 105 | 417 |
| KU720415.1 | MR766 | Uganda | 1947 | - | Vero (x3) | 10766 | 99 | 395 |
| KX087101.2 | ZIKV/Homo sapiens/PRI/PRVABC59/2015 | Peurto Rico | 2015 | *Homo sapiens* | Vero (x5) | 10764 | 73 | 420 |
| KF268950.1 [[9](#_ENREF_9)] | ARB7701 | Central African Republic | 1976 | *Aedes africanus* | - | 10755 | 106 | 380 |
| KU729218.1* [[10](#_ENREF_10)] | BeH828305 | Brazil | 2015 | *Homo sapiens* | - | 10729 | 94 | 364 |
| KU365778.1* [[10](#_ENREF_10)] | BeH819015 | Brazil | 2015 | *Homo sapiens* | - | 10727 | 94 | 361 |
| KU963573.1 | Zika virus/M.mulatta-tc/UGA/MR-766_SM150-V8/1947 | Uganda | 1947 | *Macaca mulatta* | Suckling mouse (x150), Vero (x8) | 10710 | 141 | 209 |
| KU963796.1* [[11](#_ENREF_11)] | SZ-WIV01 | China (imported from Somoa) | 2016 | *Homo sapiens* | - | 10709 | 58 | 379 |
| KU321639.1* [[12](#_ENREF_12)] | ZikaSPH2015 | Brazil | 2015 | *Homo sapiens* | - | 10676 | 105 | 299 |
| KU744693.1* [[13](#_ENREF_13)] | VE_Ganxian | China (Jiangxi), imported from Venezuela | 2016 | *Homo sapiens* | - | 10676 | 105 | 299 |
| KU501215.1* [[14](#_ENREF_14)] | PRVABC59 | Peurto Rico | 2015 | *Homo sapiens* | - | 10675 | 106 | 297 |
| KU365780.1* [[10](#_ENREF_10)] | BeH815744 | Brazil | 2015 | *Homo sapiens* | - | 10662 | 93 | 297 |
| KU365779.1* [[10](#_ENREF_10)] | BeH819966 | Brazil | 2015 | *Homo sapiens* | - | 10662 | 93 | 297 |
| KU365777.1* [[10](#_ENREF_10)] | BeH818995 | Brazil | 2015 | *Homo sapiens* | - | 10662 | 94 | 297 |
| KX087102.1 | ZIKV/Homo sapiens/COL/FLR/2015 | Colombia: Barranquilla | 2015 | *Homo sapiens* | C6/36 (x3) | 10661 | 91 | 299 |
| KU707826.1* [[15](#_ENREF_15)] | SSABR1 | Salvador, Bahia, Brazil | 2015 | *Homo sapiens* | - | 10648 | 78 | 298 |
| KU729217.2* [[10](#_ENREF_10)] | BeH823339 | Brazil | 2015 | *Homo sapiens* | - | 10645 | 75 | 298 |
| KU991811.1* | Brazil/2016/INMI1 | Italy imported from Brazil (Rio de Janeiro state) | 2016 | *Homo sapiens* | - | 10643 | 73 | 298 |
| KU853013.1* [[16](#_ENREF_16)] | Dominican Republic/2016/PD2 | Dominican republic (patient hospitalised in Italy) | 2016 | *Homo sapiens* | x1 - cell type not stated | 10636 | 107 | 257 |
| KU853012.1* [[16](#_ENREF_16)] | Dominican Republic/2016/PD1 | Dominican republic (patient hospitalised in Italy) | 2016 | *Homo sapiens (urine)* | - | 10636 | 107 | 257 |
| KJ776791.1* [[17](#_ENREF_17)] | H/PF/2013 | French Polynesia | 2013 | *Homo sapiens* | Vero | 10617 | 47 | 298 |
| KU647676.1* [[18](#_ENREF_18)] | MRS_OPY_Martinique_PaRi_2015 | Martinique | 2015 | *Homo sapiens* | - | 10617 | 47 | 298 |
| KU922960.1* | MEX/InDRE/Sm/2016 | Mexico | 2016 | *Homo sapiens* (saliva) | - | 10617 | 47 | 298 |
| KU922923.1* | Mex/InDRE/Lm/2016 | Mexico | 2016 | *Homo sapiens* (CSF) | - | 10617 | 47 | 298 |
| KU740184.2* | GD01 | China (South America to Guandong) | 2016 | *Homo sapiens* | - | 10574 | 62 | 240 |
| KU955589.1* | Z16006 | China | 2016 | *Homo sapiens* | - | 10574 | 40 | 262 |
| KU955590.1* | Z16019 | China | 2016 | *Homo sapiens* | - | 10569 | 44 | 253 |
| KU963574.1 | ZIKV/Homo sapiens/NGA/IbH-30656_SM21V1-V3/1968 | Nigeria | 1968 | *Homo sapiens* | Suckling mouse (x21), Vero (x4) | 10442 | 126 | 62 |
| KU761564.1* | GDZ16001 | China (South America to Guandong) | 2016 | *Homo sapiens* (saliva) | - | 10401 | 48 | 81 |
| KU312312.1* [[19](#_ENREF_19)] | Z1106033 | Suriname | 2015 | *Homo sapiens* | - | 10374 | 40 | 62 |
| KF383115.1 [[20](#_ENREF_20)] | ArB1362 | Central African Republic | 1968 | *Aedes africanus* | - | 10272 | - | - |
| KF383116.1 [[20](#_ENREF_20)] | ArD7117 | Senegal | 1968 | *Aedes luteocephalus* | - | 10272 | - | - |
| KF383117.1 [[20](#_ENREF_20)] | ArD128000 | Senegal | 1997 | *Aedes luteocephalus* | - | 10272 | - | - |
| KF383118.1 [[20](#_ENREF_20)] | ArD157995 | Senegal | 2001 | *Aedes dalzieli* | - | 10272 | - | - |
| KF383119.1 [[20](#_ENREF_20)] | ArD158084 | Senegal | 2001 | *Aedes dalzieli* | - | 10272 | - | - |
| EU545988.1* [[21](#_ENREF_21)] | ZIKV 2007 epidemic consensus (EC_2007) | Yap Island-Micronesia | 2007 | *Homo sapiens* | - | 10272 | - | - |
| KU501217.1* [[14](#_ENREF_14)] | 8375 | Guatemala | 2015 | *Homo sapiens* | - | 10272 | - | - |
| KU501216.1* [[14](#_ENREF_14)] | 103344 | Guatemala | 2015 | *Homo sapiens* | - | 10272 | - | - |
| KU820898.1* | GZ01 | China (South America to Guandong) | 2016 | *Homo sapiens* (urine) | - | 10272 | - | - |
| KU866423.1* [[22](#_ENREF_22)] | Zika virus/SZ01/2016 | China imported from Samoa | 2016 | *Homo sapiens* | - | 10272 | - | - |
| KX056898.1* | Zika virus/GZ02/2016 | China | 2016 | *Homo sapiens* | - | 10272 | - | - |
| DQ859059 [[23](#_ENREF_23)] | MR766 | Uganda | - | *-* | - | 10254 | - | - |
| KX377335 | MR766 | Uganda | 1947 | *Macaca mulatta* | - | 10807 | 107 | 429 |

* indicates accession numbers used to generate maximum clade credibility tree (Fig 1). ** indicates sequence which has been updated after analyses were performed. Number of passages are described as (x_).

**References**

1. Mlakar J, Korva M, Tul N, Popovic M, Poljsak-Prijatelj M, et al. (2016) Zika Virus Associated with Microcephaly. N Engl J Med 374: 951-958.

2. Ladner JT, Wiley MR, Prieto K, Yasuda CY, Nagle E, et al. (2016) Complete Genome Sequences of Five Zika Virus Isolates. Genome Announc 4.

3. Ellison DW, Ladner JT, Buathong R, Alera MT, Wiley MR, et al. (2016) Complete Genome Sequences of Zika Virus Strains Isolated from the Blood of Patients in Thailand in 2014 and the Philippines in 2012. Genome Announc 4.

4. Lednicky J, Beau De Rochars VM, El Badry M, Loeb J, Telisma T, et al. (2016) Zika Virus Outbreak in Haiti in 2014: Molecular and Clinical Data. PLoS Negl Trop Dis 10: e0004687.

5. Driggers RW, Ho CY, Korhonen EM, Kuivanen S, Jaaskelainen AJ, et al. (2016) Zika Virus Infection with Prolonged Maternal Viremia and Fetal Brain Abnormalities. N Engl J Med.

6. Kuno G, Chang GJ (2007) Full-length sequencing and genomic characterization of Bagaza, Kedougou, and Zika viruses. Arch Virol 152: 687-696.

7. Kuno G, Chang GJ (2005) Biological transmission of arboviruses: reexamination of and new insights into components, mechanisms, and unique traits as well as their evolutionary trends. Clin Microbiol Rev 18: 608-637.

8. Calvet G, Aguiar RS, Melo AS, Sampaio SA, de Filippis I, et al. (2016) Detection and sequencing of Zika virus from amniotic fluid of fetuses with microcephaly in Brazil: a case study. Lancet Infect Dis.

9. Berthet N, Nakoune E, Kamgang B, Selekon B, Descorps-Declere S, et al. (2014) Molecular characterization of three Zika flaviviruses obtained from sylvatic mosquitoes in the Central African Republic. Vector Borne Zoonotic Dis 14: 862-865.

10. Faria NR, Azevedo Rdo S, Kraemer MU, Souza R, Cunha MS, et al. (2016) Zika virus in the Americas: Early epidemiological and genetic findings. Science 352: 345-349.

11. Deng C, Liu S, Zhang Q, Xu M, Zhang H, et al. (2016) Isolation and characterization of Zika virus imported to China using C6/36 mosquito cells. Virol Sin 31: 176-179.

12. Cunha MS, Esposito DL, Rocco IM, Maeda AY, Vasami FG, et al. (2016) First Complete Genome Sequence of Zika Virus (Flaviviridae, Flavivirus) from an Autochthonous Transmission in Brazil. Genome Announc 4.

13. Liu L, Wu W, Zhao X, Xiong Y, Zhang S, et al. (2016) Complete Genome Sequence of Zika Virus from the First Imported Case in Mainland China. Genome Announc 4.

14. Lanciotti RS, Lambert AJ, Holodniy M, Saavedra S, Signor Ldel C (2016) Phylogeny of Zika Virus in Western Hemisphere, 2015. Emerg Infect Dis 22: 933-935.

15. Giovanetti M, Faria NR, Nunes MR, de Vasconcelos JM, Lourenco J, et al. (2016) Zika virus complete genome from Salvador, Bahia, Brazil. Infect Genet Evol 41: 142-145.

16. Barzon L, Pacenti M, Berto A, Sinigaglia A, Franchin E, et al. (2016) Isolation of infectious Zika virus from saliva and prolonged viral RNA shedding in a traveller returning from the Dominican Republic to Italy, January 2016. Euro Surveill 21.

17. Baronti C, Piorkowski G, Charrel RN, Boubis L, Leparc-Goffart I, et al. (2014) Complete coding sequence of zika virus from a French polynesia outbreak in 2013. Genome Announc 2.

18. Piorkowski G, Richard P, Baronti C, Gallian P, Charrel R, et al. Complete coding sequence of Zika virus from Martinique outbreak in 2015. New Microbes and New Infections 11: 52-53.

19. Enfissi A, Codrington J, Roosblad J, Kazanji M, Rousset D (2016) Zika virus genome from the Americas. Lancet 387: 227-228.

20. Faye O, Freire CC, Iamarino A, Faye O, de Oliveira JV, et al. (2014) Molecular evolution of Zika virus during its emergence in the 20(th) century. PLoS Negl Trop Dis 8: e2636.

21. Lanciotti RS, Kosoy OL, Laven JJ, Velez JO, Lambert AJ, et al. (2008) Genetic and serologic properties of Zika virus associated with an epidemic, Yap State, Micronesia, 2007. Emerg Infect Dis 14: 1232-1239.

22. Deng YQ, Zhao H, Li XF, Zhang NN, Liu ZY, et al. (2016) Isolation, identification and genomic characterization of the Asian lineage Zika virus imported to China. Sci China Life Sci 59: 428-430.

23. Grard G, Moureau G, Charrel RN, Holmes EC, Gould EA, et al. (2010) Genomics and evolution of Aedes-borne flaviviruses. J Gen Virol 91: 87-94.
