# Supplementary material for: Full Genome Sequence and sfRNA Interferon Antagonist Activity of Zika Virus from Recife, Brazil
Source: PLoS Negl Trop Dis. 2016 Oct 5;10(10):e0005048. doi: 10.1371/journal.pntd.0005048 (PMC5051680; doi:10.1371/journal.pntd.0005048)
Supplement: S2 Table — Positions of single nucleotide mutations within the predicted sfRNA sequences of three African lineage isolates compared to ZIKV PE243 sfRNA. Mutations are described as Asian lineage: African lineage. * indicates MR766 conserved mutations. (DOCX) [file pntd.0005048.s002.docx]

| **Mutation (position relative to start of 3’ UTR)** | **Associated secondary structure** |
| --- | --- |
| C9U | - |
| A13G* | - |
| C42U | SLII (matching partner for G52) |
| G66A | SLII (matching partner for U) |
| C97U | - |
| U98C | - |
| G192A | - |
| U257C* | - |
| C258U | - |
| G266A | - |
| A275C | - |
| U395C | Loop forming flexible region |
| G425U* | - |
| C427U* | - |
| U428C* | - |
| - 429U* | - |
