# Supplementary figures and images for: Full Genome Sequence and sfRNA Interferon Antagonist Activity of Zika Virus from Recife, Brazil
Source: PLoS Negl Trop Dis. 2016 Oct 5;10(10):e0005048. doi: 10.1371/journal.pntd.0005048 (PMC5051680; doi:10.1371/journal.pntd.0005048)

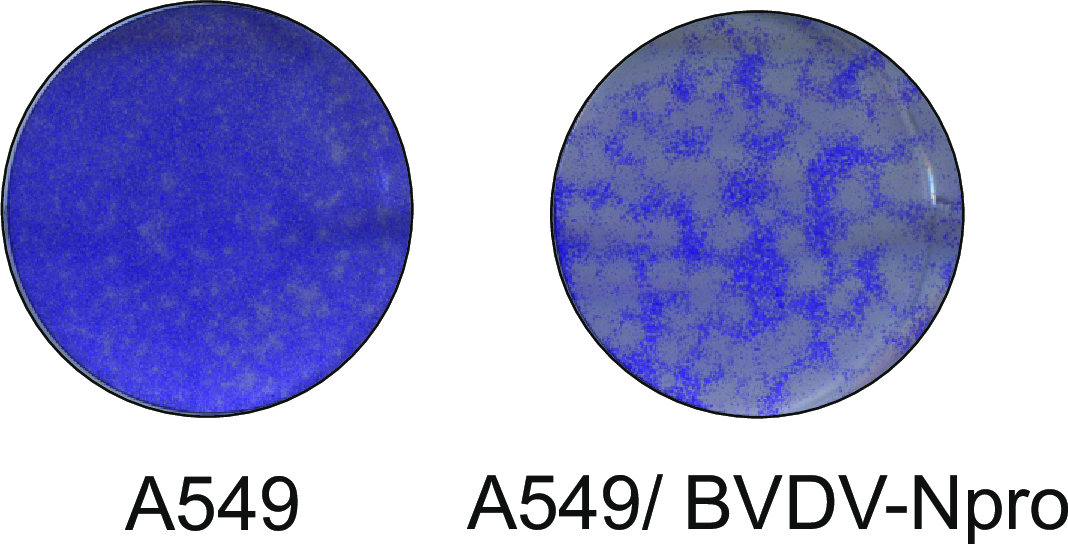

Supplement: S1 Fig — Virus growth was analyzed by plaque size comparisons in human A549 (interferon competent) and A549/BVDV-Npro (type I interferon incompetent) cell lines. (TIF) [file pntd.0005048.s003.tif]

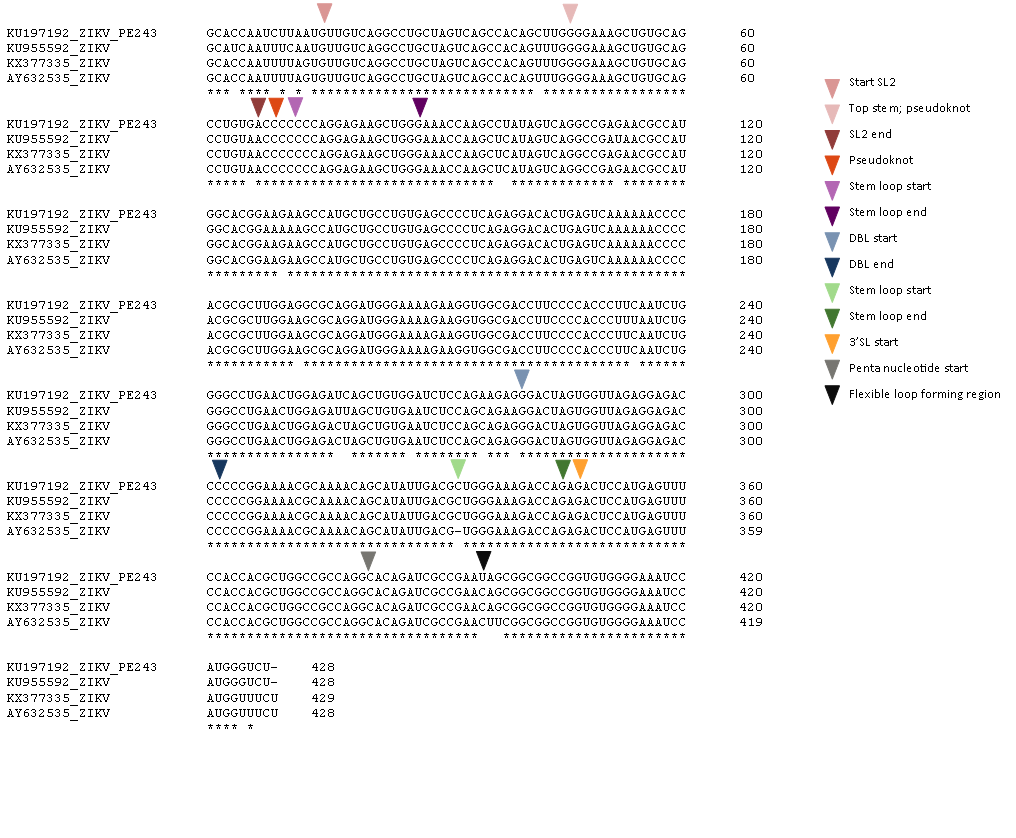

Supplement: S2 Fig — Accession numbers African ZIKV: MR766 isolates AY632535 and KX377335; further strain KU955592. Predicted sequence elements and structures are indicated. (DOCX) [file pntd.0005048.s004.docx]

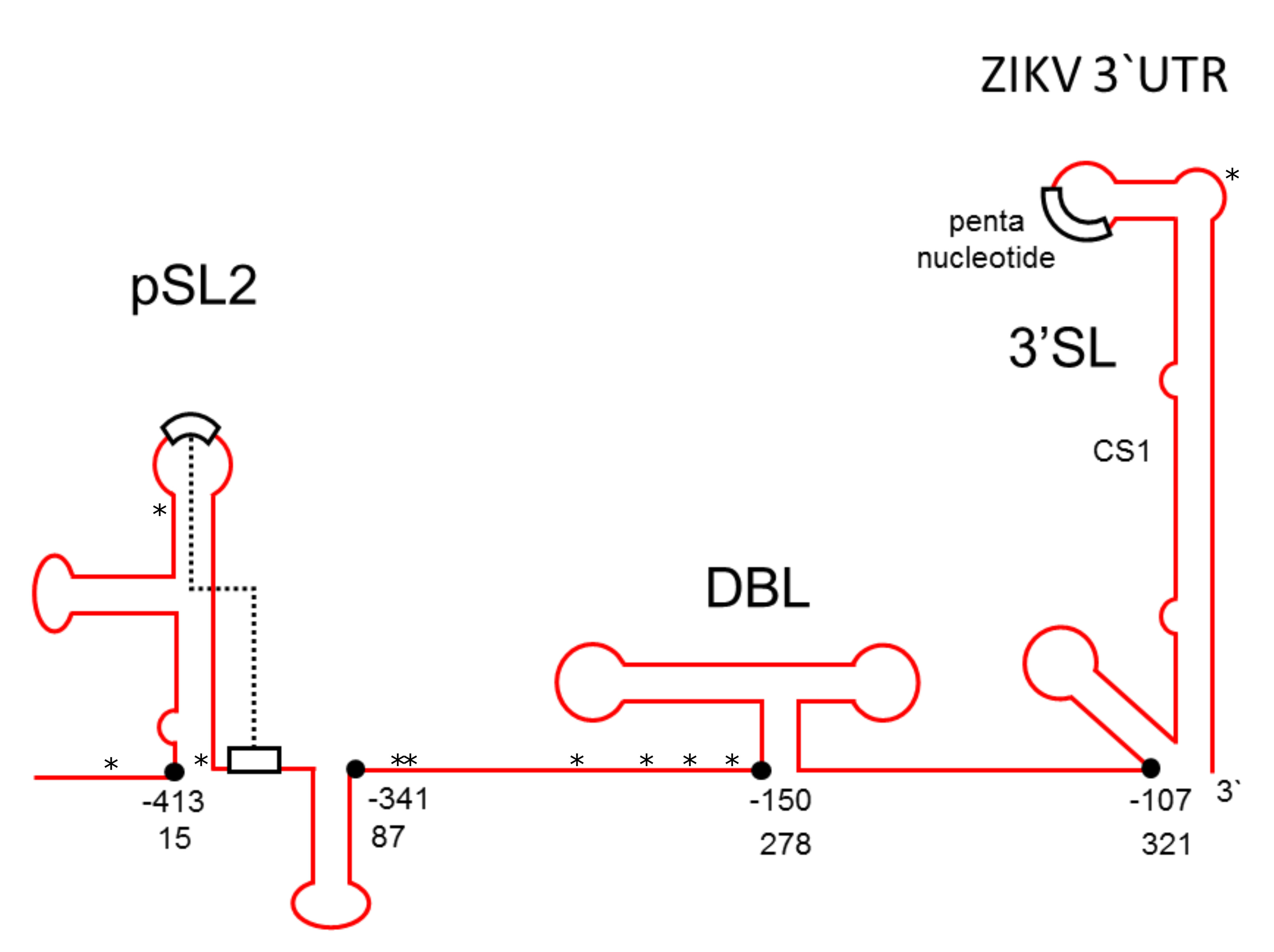

Supplement: S3 Fig — Shown is 5’-3’ of the ZIKV PE243 3’UTR sequence, left to right (as also shown in Fig 5). Asterisks indicate conserved mutations between all compared African lineage sequences (isolates AY632535, KX377335 and KU955592) and location in the predicted ZIKV PE243 sfRNA. (TIF) [file pntd.0005048.s005.tif]
