# Supplementary material for: Full Genome Sequence and sfRNA Interferon Antagonist Activity of Zika Virus from Recife, Brazil
Source: PLoS Negl Trop Dis. 2016 Oct 5;10(10):e0005048. doi: 10.1371/journal.pntd.0005048 (PMC5051680; doi:10.1371/journal.pntd.0005048)
Supplement: S1 File — (DOCX) [file pntd.0005048.s006.docx]

**Supplemental Information**

**1. Use of mouse anti-ZIKV serum**

The ZIKV PE243 isolate was initially analysed by indirect immunofluorescence assay (IFA). Vero E6 cells were infected with ZIKV at an MOI of 1 and fixed 24 h p.i. with 8% formaldehyde, followed by immunostaining with anti-ZIKV mouse antibodies diluted 1:100 in 2% FBS in PBS. Detection of bound primary antibodies was achieved using an anti-mouse secondary antibody conjugated to AlexaFluor® 488 (ThermoFisher Scientific) diluted 1:250 in 2% FBS in PBS. Images were analysed using a Zeiss LSM 710 inverted confocal microscope.  A ZIKV-specific signal was detected in virus-infected cells (Fig 1).


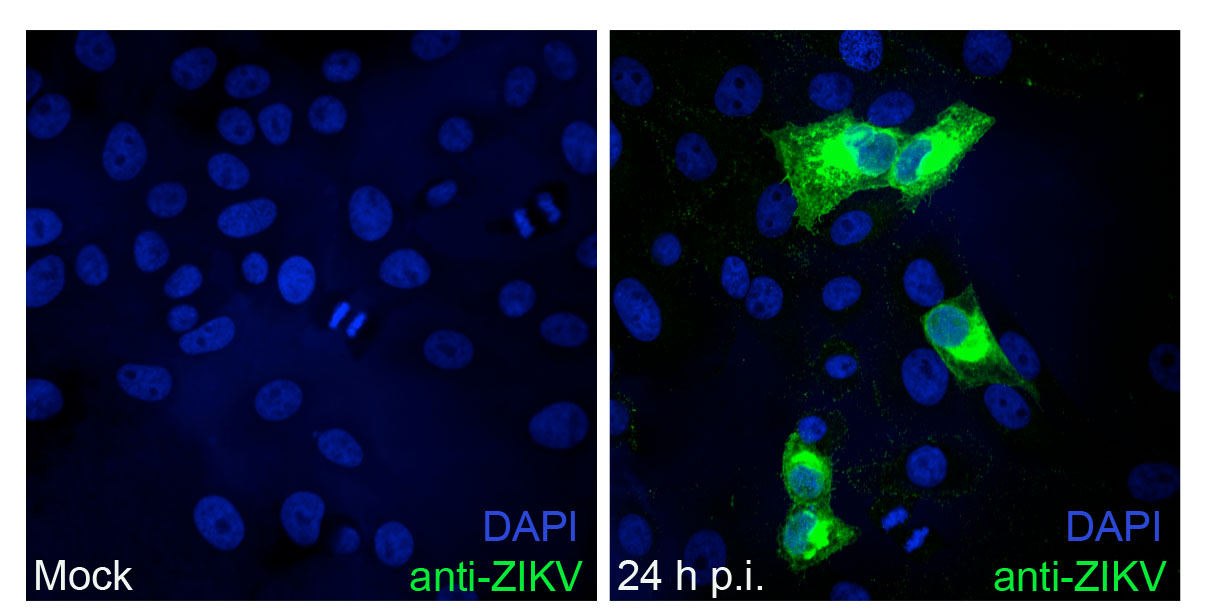


**Fig 1.** **Detection of ZIKV by using mouse anti-ZIKV serum and IFA.** Mock infected (left panel) or ZIKV infected (MOI 1, right panel) Vero E6 cells were fixed 24 h p.i. Immunofluorescence analysis was performed by using mouse anti-ZIKV serum.

**2. Use of E antibodies**

A panel of antibodies against ZIKV E protein was purchased from Aalto Bio Reagents (Ireland). The ZIKV PE243 isolate was further analysed by IFA and western blot analysis by using commercially available antibodies against the ZIKV E protein (Table 1). Western blot analysis of total cell lysate utilizing ZIKV_E_ Ab1 and ZIKV_E_ Ab3 antibodies, generated two bands; a higher molecular weight band observed between 48- 68 kDa corresponding in molecular weight to the predicted ZIKV E protein (54 kDa), and a lower molecular weight band between 38- 48 kDa (Fig 2). ZIKV_E_Ab2 gave only one band at the expected molecular weight for E. All antibodies gave a highly specific signal by IFA in ZIKV-infected cells. These antibodies therefore appear suitable for future studies of ZIKV.

**Immunofluorescence and confocal imaging**

Vero E6 cells were infected with ZIKV at an MOI of 1 and fixed 48 h p.i. with 8% formaldehyde, followed by immunostaining for ZIKV E with mouse IgG antibodies (Aalto Bio Reagents) diluted in 5% FBS in PBS as described in Table 1. Detection of bound primary antibodies was achieved using an anti-mouse secondary antibody conjugated to AlexaFluor® 488 (ThermoFisher Scientific) diluted 1:1,000 in 5% FBS in PBS. Images were analysed using a Zeiss LSM 710 inverted confocal microscope.

**Western blotting**

SDS-PAGE and western blots were performed using lysates from Vero E6 cells infected with ZIKV at an MOI of 3 that were harvested at 48 h p.i. Samples were run on a 4%-12% gradient Bis Tris gel (Life Technologies) and blotted to a nitrocellulose membrane (GE Healthcare). Protein expression was determined using ZIKV specific antibodies described in Table 1 (Aalto Bio Reagents) diluted in PBS with 5% dried milk and 0.1% Tween 20. Secondary detection was achieved using an anti-mouse antibody conjugated to HRP (Life Technologies) and visualised by enhanced chemiluminescence using Pierce ECL western blot substrate (Thermo Scientific) as per the manufacturer’s instructions.

**Table 1. ZIKV primary antibodies for detection of ZIKV E protein by western blotting and immunofluorescence analysis.**

| **Designated Name** | **Product code** | **Western blot**  **working ratio** | **Immunofluorescence**  **working ratio** |
| --- | --- | --- | --- |
| ZIKV_E_Ab1 | AZ1176/0302156 | 1:10000 | 1:500 |
| ZIKV_E_Ab2 | AZ1176/0402166 | 1:5000 | 1:400 |
| ZIKV_E_Ab3 | AZ1176/0502176 | 1:10000 | 1:1000 |


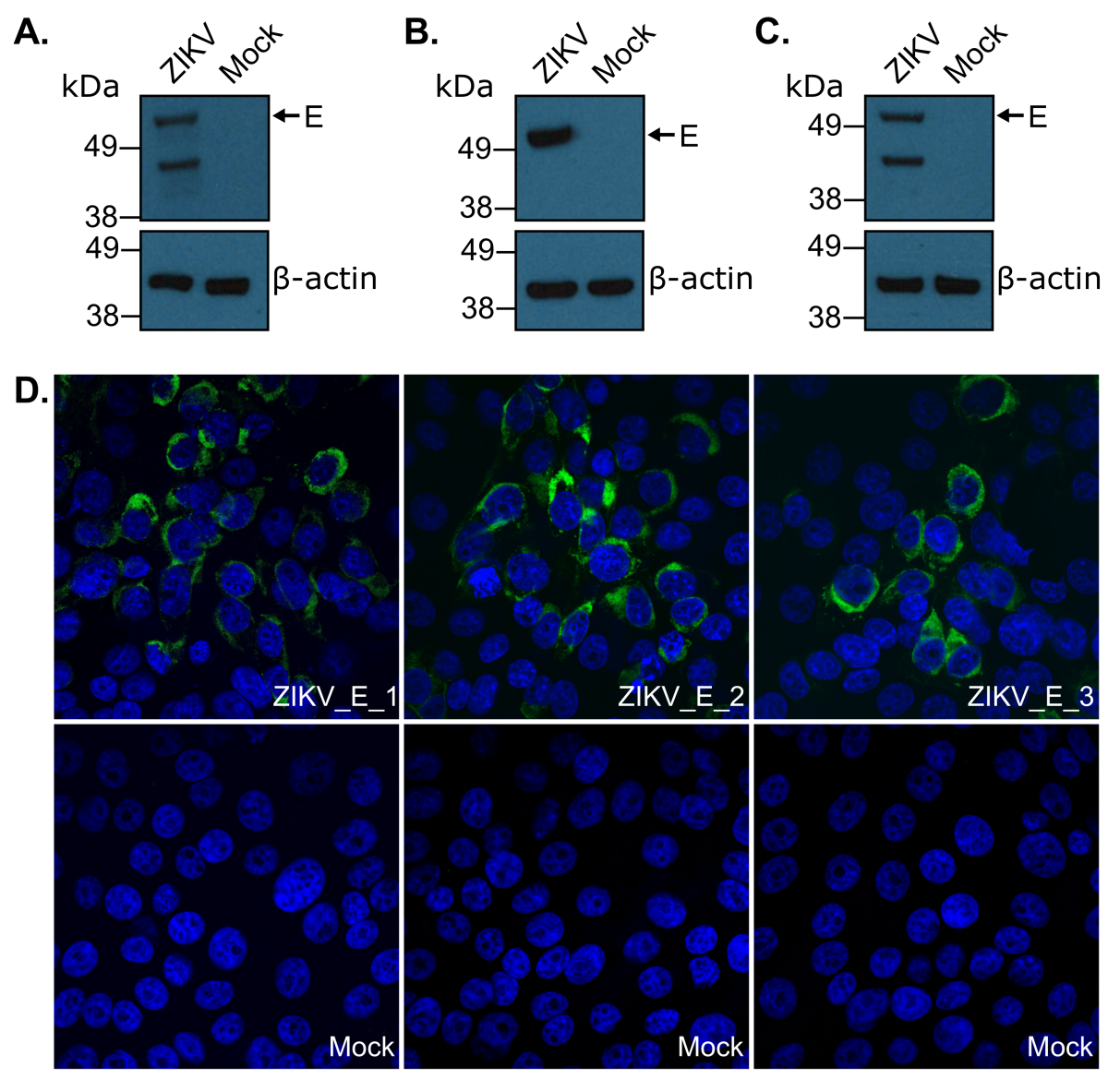


**Fig 2.** **Characterization of commercially available anti-ZIKV E protein monoclonal antibodies.** ZIKV-infected (MOI 3) and mock-infected Vero E6 cells were lysed 48 h p.i and analyzed by western blot. Detection of ZIKV E was achieved using ZIKV_E_Ab1 (**A**) ZIKV_E_Ab2 (**B**) or ZIKV_E_Ab3 (**C**) (Table 1). **(D**) ZIKV infected (MOI 1, top panels) and mock infected (bottom panels) Vero E6 cells were fixed 48 h p.i. and immunofluorescence analysis performed using ZIKV_E_Ab1 (labelled E_1), ZIKV_E_Ab2 (labelled E_2) or ZIKV_E_Ab3 (labelled E_3) antibodies (labelled E_3) (see Table 1 for details).
